# Phosphoenolpyruvate Carboxykinase 1 Gene (*Pck1*) Displays Parallel Evolution between Old World and New World Fruit Bats

**DOI:** 10.1371/journal.pone.0118666

**Published:** 2015-03-25

**Authors:** Lei Zhu, Qiuyuan Yin, David M. Irwin, Shuyi Zhang

**Affiliations:** 1 Institute of Molecular Ecology and Evolution, SKLEC & IECR & IAIR, East China Normal University, Shanghai, China; 2 Department of Laboratory Medicine and Pathobiology, University of Toronto, Toronto, Canada; University of Lausanne, SWITZERLAND

## Abstract

Bats are an ideal mammalian group for exploring adaptations to fasting due to their large variety of diets and because fasting is a regular part of their life cycle. Mammals fed on a carbohydrate-rich diet experience a rapid decrease in blood glucose levels during a fast, thus, the development of mechanisms to resist the consequences of regular fasts, experienced on a daily basis, must have been crucial in the evolution of frugivorous bats. Phosphoenolpyruvate carboxykinase 1 (PEPCK1, encoded by the *Pck1* gene) is the rate-limiting enzyme in gluconeogenesis and is largely responsible for the maintenance of glucose homeostasis during fasting in fruit-eating bats. To test whether *Pck1* has experienced adaptive evolution in frugivorous bats, we obtained *Pck1* coding sequence from 20 species of bats, including five Old World fruit bats (OWFBs) (Pteropodidae) and two New World fruit bats (NWFBs) (Phyllostomidae). Our molecular evolutionary analyses of these sequences revealed that *Pck1* was under purifying selection in both Old World and New World fruit bats with no evidence of positive selection detected in either ancestral branch leading to fruit bats. Interestingly, however, six specific amino acid substitutions were detected on the ancestral lineage of OWFBs. In addition, we found considerable evidence for parallel evolution, at the amino acid level, between the PEPCK1 sequences of Old World fruit bats and New World fruit bats. Test for parallel evolution showed that four parallel substitutions (Q276R, R503H, I558V and Q593R) were driven by natural selection. Our study provides evidence that *Pck1* underwent parallel evolution between Old World and New World fruit bats, two lineages of mammals that feed on a carbohydrate-rich diet and experience regular periods of fasting as part of their life cycle.

## Introduction

Food limitation is a common physiological challenge faced by animals and leads to a risk of death due to starvation. Prolonged fasting, and starvation, causes a reduction in blood glucose levels, which can result in hypoglycemia and threaten life [1,2]. In this study, we focused on the molecular evolution of a gene involved in the resistance to the consequences of fasting. Maintaining glucose homeostasis under fasting conditions is essential [3], and many tissues are known to be involved in the regulation of blood glucose levels [4]. The liver plays a major role, as hepatic gluconeogenesis is the primary metabolic mechanism in the fasting state, in generating fuel to maintain the basic functions of other tissues, including skeletal muscle, red blood cells, and the brain [5]. In addition to the liver, renal and intestinal gluconeogeneses also contribute to the maintenance of glucose homeostasis during fasting [6,7]. The gluconeogenic pathway contains 11 steps that are catalyzed in turn by 11 enzymes [8] (S1 Fig.). The enzyme phosphoenolpyruvate carboxykinase (PEPCK), which catalyzes an irreversible reaction, is the rate-limiting step in gluconeogenesis [9]. Mammals have two isozymes of PEPCK, which are encoded by separate genes: phosphoenolpyruvate carboxykinase1 (PEPCK1, encoded by the *Pck1* gene), which is located in the cytosol, and phosphoenolpyruvate carboxykinase 2 (PEPCK2, encoded by the *Pck2* gene), which is restricted to the mitochondria [10]. PEPCK1 accounts for about 95% of the PEPCK enzymatic activity in rats and mice, which are popular model organisms [11,12], and its single copy gene consisting of 10 exons [13]. Studies in *Pck1*-knockout mice confirmed the importance of *Pck1* in hepatic gluconeogenesis and glucose homeostasis, as *Pck1* ^-/-^ mice display severe hypoglycemia [14]. The level of transcription of PEPCK1 determines the rate of gluconeogenesis [15].

Mammals consume a variety of different diets, which has led to the development of differing approaches to store and utilize body energy reserves, with different responses to fasting evolving in different species [16]. Previous studies have shown that the plasma glucose levels of animals fed a carbohydrate-rich diets fall severely during fasting, which contrasts to the steady blood glucose concentrations observed in vertebrates fed a protein-rich diet [17–19], suggesting that animals with a carbohydrate-rich diet should be less capable of resisting the consequences of a fast.

Among mammalian orders, bats have the widest range of diets, with diets based on a variety of specific food sources including insects, large arthropods, small vertebrates, blood, nectar, fruits, or leaves [20,21]. Old World fruit bats (OWFB) and New World fruit bats (NWFB), belonging to the families Pteropodidae and Phyllostomidae, respectively, have independently acquired carbohydrate-rich diets that are comprised mostly of fruit and/or nectar [22,23]. Bats dedicate only a very small portion (up to 4 h) of their daily activity cycle to foraging, and spend the remainder of their day in sheltered roosts, where they neither eat nor drink [24]. Therefore, bats experience both short and long term fasts as regular occurrences [25]. The evolution of flight in bats has also brought high energetic costs, as bats in flight have metabolic rates about 15 times greater than those at rest [26–29]. Since bats in flight have extremely high metabolic rates, one might expect that bats that feed on carbohydrate rich diets should show a greater consequence to fasting than other bats. Glucose homeostasis during a fast can be maintained by two primary mechanisms: glycogenolysis and gluconeogenesis [8]. Recent studies have demonstrated that in frugivorous bats that gluconeogenesis, rather than glycogenolysis, is the main mechanism for regulating glucose homeostasis during a fast [30,31]. These observations make it reasonable to hypothesize that key genes involved in gluconeogenesis, through adaptations for fasting, may have undergone adaptive evolution in the fruit eating bats. The enzymatic activity levels of all key enzymes in gluconeogenesis, including Glucose-6-phosphatase, fructose-1,6-bisphosphatase, PEPCK1 and PEPCK2, were measured in two species of fruit bats (*Artibeus lituratus* and *Artibeus jamaicensis*) before and after a 24 hour fasts by Pinheiro et al [32], who concluded that a significant change in the activity of only PEPCK1 could be demonstrated. These results indicated that PEPCK1 likely has a crucial role in the adaptation of gluconeogenesis for fasting in fruit bats.

We speculated that *Pck1* experienced adaptive evolution in OWFBs and NWFBs in association with their acquired high carbohydrate diets. To test our hypothesis, we obtained the coding region of *Pck1* from 20 species of bats (including five OWFBs species and two NWFBs species), and assessed whether these sequences display molecular evolutionary patterns consistent with adaptive evolution. In addition, we also examined whether the *Pck1* gene has experienced convergent and/or parallel evolution between the OWFBs and NWFBs at the amino acid level.

## Materials and Methods

### Ethics statement

Protocols, including bat species collection and tissue sampling in China, were approved by the Animal Ethics Committee of East China Normal University (ID No: AR2012/03001). We captured *Cynopterus sphinx* from Zhongshan park (21°28′N, 109°7′E) in Guangxi province in Nov. 2009, and *Rousettus leschenaultii* were captured during Nov. 2009 from Jinlun cave (23°33′N, 108°15′E) located in Guangxi province, China. *Eonycteris spelaea* was captured in Mar. 2009 from Mengla park (21°55′N, 101°15′E) located in Yunnan province, China. *Rhinolophus pusillus* and *Rhinolophus ferrumequinum* were captured in Apr. 2013 from Lianhua cave (36°50′N, 113°54′E) located in Hebei province, China. *Hipposideros armiger* was captured in Apr. 2013 from Shihua cave (30°5′N, 119°5′E) located in Zhejiang province, China. *Hipposideros pratti* was captured in Apr. 2013 from Tianzidi cave (29°46′N, 119°21′E) located in Zhejiang province, China. *Miniopterus schreibersi* was captured in Apr. 2013 from Yunhua cave (33°18′N, 111°25′E) located in Henan province, China. *Myotis ricketti* was captured in Nov. 2012 from a cave (28°4′N, 116°58′E) located Jiangxi province, China. *Pipistrellus abramus* was captured in Jul. 2011 from a park (23°26′N, 111°30′E) located in Jiangxi province, China. *Ia io* was captured in Aug. 2013 near Xilin road (24°23′N, 106°17′E) located in Yunnan province, China. *Scotophilus heathi* was captured in Jul. 2011 from a building (23°1′N, 114°56′E) located in Guangdong province, China. *Artibeus lituratus*, *Leptonycteris yerbabuenae* and *Pteronotus parnellii* were captured in Apr. 2010 in Mexico. Bats captured in these locations were permitted by the local Protection and Research Center. No bat species used for this study are considered endangered. Bats were sacrificed by decapitation, quickly after capture, and all efforts were made to minimize potential pain and suffering.

### Taxonomic coverage

In this study, we sequenced the coding region (97%) of the *Pck1* gene from 15 species of bats, which were then combined with sequences from five other bat species obtained from NCBI and Ensembl. Our dataset included sequences from the following species. From the suborder Yinpterochiroptera, we included five OWFBs from the family pteropodidae (*Pteropusvampyrus*, *Pteropus alecto*, *Cynopterus sphinx*, *Eonycteris spelaea*, and *Rousettus leschenaultii*) and four insectivorous species from sister families to OWFBs (*Rhinolophus pusillus*, *Rhinolophus ferrumequinum*, *Hipposideros armiger* and *Hipposideros pratti*). From the suborder Yangochiroptera, we sampled two NWFBs from the family Phyllostomidae (*Artibeus lituratus* and *Leptonycteris yerbabuenae*) and nine insectivorous bat species (*Pteronotus parnellii*, *Miniopterus schreibersi*, *Scotophilus heathi*, *Ia io*, *Pipistrellus abramus*, *Myotis ricketti*, *Myotis davidii*, *Myotis brandtii*, and *Myotis lucifugus*). All new *Pck1* sequences were deposited into GenBank with the following accession numbers: KJ957749-KJ957763.

To expand our dataset across mammals we incorporated published *Pck1* sequences from nine other mammalian species from NCBI and Ensembl: *Homo sapiens* (NM_002591), *Mus musculus* (NM_011044), *Rattus norvegicus* (NM_198780), *Equus caballus* (XM_001489771), *Bos taurus* (NM_174737), *Sus scrofa* (NM_001123158), *Canis familiaris* (NM_001197143), *Loxodonta Africana* (XM_003419943), and *Monodelphis domestica* (XM_001377770). Details on all species and their corresponding accession numbers are listed in S1 Table.

### Isolation, Amplification and Sequencing of Pck1 Coding Sequences

For the 15 bat species used to isolate new sequences, total RNA was isolated from liver tissue (stored at -80°C) using the Trizol reagent. Following the standard protocol, 5 μg of total RNA was reverse transcribed into cDNA using the Reverse Transcriptase kit. Primers (F: ATGCCTCCTCAGCTGCAAAACG and R: TACATCTGGCTGACTCTCTGCCTC) were designed to amplify the coding sequences of *Pck1*. All PCR products were separated using 1% agarose gels, with the correct sized fragments purified with Gel Extraction Kits, ligated into pGEM-T easy vector, cloned, and sequenced using Terminator kits on an ABI 3730 DNA sequencer. To avoid artifacts, multiple clones (>5) were sequenced for each species.

### Phylogenetic Reconstruction

*Pck1* nucleotide sequences from 29 mammalian species were aligned using ClustalX [33] and checked for accuracy by eye, with the coding sequences translated into amino acids using MEGA5 [34]. A Bayesian phylogenetic tree was reconstructed based on the aligned nucleotide sequences using MrBayes 3.1.2 [35] with the GTR+I+Γ nucleotide substitution model selected as the best-fitting model by jModelTest0.1 [36]. For the Bayesian analysis, we performed 10,000,000 generations of MCMC and sampled every 100 generations, with the first 2,000,000 generations discarded as burn-in. All other options and priors were the default settings of the MrBayes 3.1.2 software. The standard deviations of split frequencies were stably below 0.01 after 2,000,000 generations of MCMC performances.

### Molecular evolution analyses

Before beginning the molecular evolution analyses, we first tested the sequences for evidence of recombination, since recombination may lead to the incorrect results in tests for positive selection. We used GARD [37] from the HyPhy package [38] to detect evidence for recombination breakpoints and tested their statistical significance. For tests of selection we used a topology for the phylogenetic tree that was based on the accepted species relationships [39–42]. For positive selection in *Pck1*, we estimated the rates of nonsynonymous (d_N_) and synonymous (d_S_) substitutions using CODEML from the PAML package [43]. We first used the two-ratio model, where the d_N_/d_S_ ratio (termed omega or ω) was allowed to be different on the foreground and background branches. To detect differences in the selective pressure acting on the ancestral branches leading to OWFBs and NWFBs, each of these two groups were separately set as the foreground branch. The one ratio-model, where the omega values were equal among branches, served as the null hypothesis for these comparisons. We also employed the free-ratio model, where the value of d_N_/d_S_ varies among all branches, to explore the overall selection pressure acting on *Pck1* in the 29 mammalian species used in this study [44]. Similarly, the one-ratio model served as the null expectation for this comparison. “PairwiseRelativeRate.bf” from the HyPhy package [38] was also used to detect whether there were significant differences in the rates of synonymous and nonsynonymous substitutions between the frugivorous and insectivorous bats (see S1 Text for details).

The branch-site model A (test1 and test2) was then applied to detect positively selected changes along particular branches [45]. In this model, the branch of interest is set as the foreground, with the remaining branches set as the background. We conducted both test1 and test2 of the branch-site models [46] on the ancestral branches for the OWFBs and NWFBs. In both tests, the branch-site model A is the alternative hypothesis. In test1, the null hypothesis is the M1a (Nearly Neutral) model. For test2, the null hypothesis is the branch-site null model. Results from the alternative and null hypothesis were then compared in likelihood ratio tests (LRTs).

Ancestral sequences were reconstructed using CODEML from the PAML package [47], and the amino acid substitutions on each branch were then inferred. The identity and direction of the substitutions was also assessed using the maximum parsimony method with Mesquite 2.74 [48]. To further explore the heterogeneous selection pressure acting across codons of the *Pck1* gene in frugivorous bats, a sliding window analysis of the ω values was performed using the program SWAAP 1.0.2 [49] with the Nei and Gojobori method [50]. Window and step sizes for this analysis were set as 90 and 36 nucleotides, respectively.

To detect evidence of parallel or convergent amino acid substitutions between OWFBs and NWFBs, we used a method described by Castoe et al. implemented in the codeMLancestral package [51] (see S1 Text). To test whether the convergence detected between pairs of focal branches was significant, we used a method described by Rossiter et al [52] (see S1 Text). We also used CONVERGE2 [53] to test whether the number of observed parallel or convergent amino acid substitutions shared by the OWFBs and NWFBs branches were significantly different from those expected by random chance. Finally, we examined whether the locations of the positively selected, convergently evolved and other specific sites in the protein sequences were associated with particular functional domains. The protein domains were defined from previous research [54,55].

## Results

Our *Pck1* sequence dataset contained 29 taxa, including five Old World fruit bats (Pteropodidae), two New World fruit bats (Phyllostomidae) and 13 insectivorous bats. The alignment of the *Pck1* coding sequence spanned 1818 nucleotides (representing ~97% of the complete coding sequence) and equates to 606 amino acids. No insertions, deletions or stop codons were identified in the isolated coding sequences from the 20 bat species, and no evidence for recombination breakpoints were detected in our dataset.

**Fig 1:**
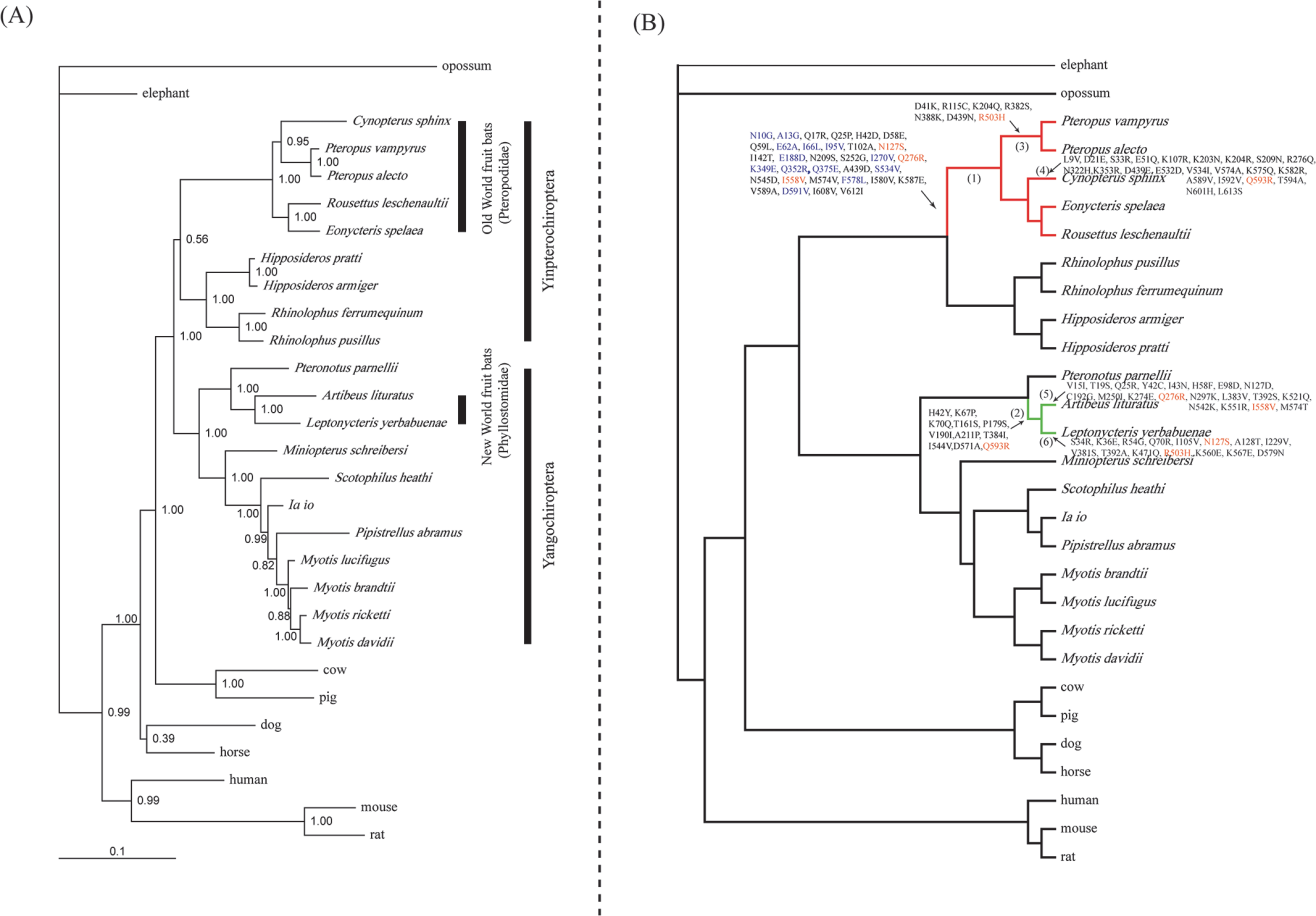
Unconstrained *Pck1* Bayesian phylogenetic tree and the species tree. (A) Unconstrained Bayesian phylogenetic tree based on the *Pck1* coding sequences, under the GTR+I+Γ model. Values on the nodes are posterior probabilities. (B) Species tree of the 29 mammals used in this study based on their accepted relationships. Red and green thick lines labeled ‘OWFBs’ and ‘NWFBs’ represent the ancestral branches for the Old World fruit bats and New World fruit bats, respectively. Nonsynonymous amino acid substitutions were mapped onto select branches. 13 sites on the ancestral branch of Old World fruit bats having omega values >1 are shown in blue. Convergent substitutions between OWFBs and NWFBs are shown in orange.

Bayesian phylogenetic reconstruction of the nucleotides sequences of *Pck1* recovered a tree with the main groupings of species in agreement with the accepted species topology [39–41] (Fig. 1B). The five OWFB species (*P*. *vampyrus*, *P*. *alecto*, *C*. *sphinx*, *E*. *spelaea*, and *R*. *leschenaultii*) grouped with the species from the family Rhinolophidae (*R*. *pusillus* and *R*. *ferrumequinum*) and Hipposideridae (*H*. *armiger* and *H*. *pratti*) to comprise the suborder Yinpterochiroptera [Bayesian posterior probability (BPP) = 0.56] (Fig. 1A). The other bat species, including the NWFBs (*A*. *lituratus* and *L*. *yerbabuenae*), grouped together to comprise the suborder Yangochiroptera (BPP = 1.00) (Fig. 1A). The two species of NWFBs grouped together by a high nodal support (BPP = 1.00) (Fig. 1A).

To detect evidence for changes in the selection pressure acting on *Pck1* in OWFBs and NWFBs, we set either the ancestral branch to OWFBs or NWFBs as the foreground branch and performed two-ratio model tests on these branches. Our results showed that the two-ratio model with the ancestral branch to OWFBs as the foreground was a better fit than the one-ratio model (null hypothesis) [likelihood ratio test (LRT) statistic (2Δ*ℓ*) = 3.99, df = 1, *P* = 0.046] (Table 1), and had an omega value (d_N_/d_S_) on the ancestral branch leading to OWFBs that was higher than on the background (0.1090 vs. 0.0676, respectively). These results indicate that a change in selection pressure on the *Pck1* gene occurred on the OWFB lineage. However, the two-ratio model test with the ancestral branch for NWFBs as the foreground was not significantly different from the one-ratio model (2Δ*ℓ* = 3.09, df = 1, *P* = 0.079) (Table 1), which implied that no change in selection pressure occurred on this NWFB lineage.

**Table 1:** Result of the branch model tests of selection pressure in the *Pck1* gene of bats.

**Model**	**np**	***ℓ***	**ω**_0_ ^a^	**ω**_OW_ ^a^	**ω**_NW_ ^a^	**Model Compare**	**2Δ*ℓ***	***P***
A. One ratio:ω_0 =_ ω_OW =_ ω_NW_	57	-13778.82	0.069	= ω_0_	= ω_0_			
B. Two ratio:ω_0 =_ ω_NW,_ω_OW_	58	-13776.83	0.068	0.109	= ω_0_	B vs. A	3.99	0.046
C. Two ratio:ω_0 =_ ω_OW,_ω_NW_	58	-13777.28	0.068	= ω_0_	0.163	C vs. A	3.09	0.079
D. Free ratio	111	-13718.94	-	-	-	D vs. A	119.77	<0.01

^a^ω_OW,_ω_NW,_ω_0,_ are the ω ratios for the OWFB, NWFB, and other branches, respectively.

Pairwise relative rate tests were also conducted to determine whether there were significant differences in the rates of synonymous and nonsynonymous substitutions between frugivorous and insectivorous bats. Our results showed that a significant difference in the rate of nonsynonymous substitutions occurs between OWFBs and insectivorous bats; however, the same result was not found for synonymous substitutions. These results from the pairwise relative rate tests indicated that a change in selection pressure on the *Pck1* gene occurred in OWFBs, and that this change mainly effected the rate of nonsynonymous substitutions (Table 2).

**Table 2:** Results of the pairwise relative rate tests.

Group 1^a^	Group 2^b^	Syn rate test^c^		Nonsyn rate test^c^		
(Old World fruit bats)	(insectivorous bats)	LRT^d^	*P*-value	LRT^d^	*p*-value
*Pteropus vampyrus*	*Rhinolophus ferrumequinum*	1.236	0.266	12.352	<0.001	
	*Hipposideros armiger*	1.201	0.273	12.733	<0.001	
	*Miniopterus schreibersi*	0.911	0.339	6.769	<0.01	
	*Pteronotus parnellii*	2.927	0.087	9.368	<0.001	
	*Myotis lucifugus*	0.442	0.506	6.586	0.010	
*Pteropus alecto*	*Rhinolophus ferrumequinum*	0.344	0.558	12.628	<0.001	
	*Hipposideros armiger*	0.316	0.574	13.430	<0.001	
	*Miniopterus schreibersi*	0.192	0.661	6.954	<0.01	
	*Pteronotus parnellii*	1.504	0.220	9.709	0.002	
	*Myotis lucifugus*	0.028	0.867	6.929	<0.01	
*Cynopterus sphinx*	*Rhinolophus ferrumequinum*	9.055	0.003	16.261	<0.001	
	*Hipposideros armiger*	60969	0.008	16.124	<0.001	
	*Miniopterus schreibersi*	6.603	0.010	9.409	0.002	
	*Pteronotus parnellii*	10.382	0.001	12.042	<0.001	
	*Myotis lucifugus*	6.017	0.014	9.449	0.002	
*Eonycteris spelaea*	*Rhinolophus ferrumequinum*	1.972	0.160	8.913	0.003	
	*Hipposideros armiger*	1.187	0.276	9.148	0.002	
	*Miniopterus schreibersi*	1.660	0.198	4.156	0.041	
	*Pteronotus parnellii*	3.119	0.077	6.879	<0.01	
	*Myotis lucifugus*	1.003	0.317	4.260	0.039	
*Rousettus leschenaultii*	*Rhinolophus ferrumequinum*	5.265	0.022	10.558	0.001	
	*Hipposideros armiger*	4.316	0.038	10.847	<0.001	
	*Miniopterus schreibersi*	4.321	0.038	5.290	0.021	
	*Pteronotus parnellii*	7.264	0.007	8.402	0.004	
	*Myotis lucifugus*	3.527	0.060	5.318	0.021	

^a^Five Old World fruit bats are used as ingroup 1 to separately compare with the insectivorous bats.

^b^Five typical Insectivorous bats are used separately as ingroup 2 for the relative rate tests to compare with the Old World fruit bats.

^c^Syn rate test, Synonymous rate tests. Nonsyn rate test, Nonsynonymous rate tests.

^d^LRT, Likelihood Ratio Test.

Branch-site model A test was performed to detect evidence for positive selection on the ancestral branches of OWFBs and NWFBs. The result of branch-site model A revealed 13 sites on the ancestral branch of OWFBs with elevated omega values (10G, 13G, 62A, 66L, 95V, 188D, 270V, 349E, 352R, 375E, 534V, 578L and 591V, amino acid positions refer to the human PEPCK1 sequence), and the fit of branch-site model A was better than the M1a model (2Δ*ℓ* = 7.95, df = 2, *P* = 0.0188; test1) (Table 3). However, the in-depth test for positive selection (test2) did not reveal a similar result, as the branch-site model A of OWFBs was not significantly better than the null branch-site null model (2Δ*ℓ* = 0.0, df = 1, *P* = 1.0; test2) (Table 3). Since test1 cannot distinguish relaxed constraints from positive selection [46], we were unable to rule out the possibility of relaxed evolution. No evidence for a change in selection pressure was found in the ancestral branch of NWFBs with both test1 and test2, as the alternative hypothesis of both the M1a model and branch-site null model were not significantly better than the null models.

**Table 3:** Result of the branch-site model A test for the detection of positively selected sites on the ancestral branches of Old World fruit bats and New World fruit bats.

Branch-site model	np^a^	Parameters	LRT^b^	***ℓ***	***P***-value	Site with elevated omega values^c^
M1a(nearly Neutral)	58	*P*_0_ = 0.906,*P*_1_ = 0.094, ω_0_ = 0.038, ω_1_ = 1.00		-13500.24		
Model A (alternative hypothesis) for Old World fruit bats	60	*P*_0_ = 0.862, *P*_1_ = 0.090, *P*_2a_ = 0.044, *P*_2b_ = 0.005 Background: ω_0_ = 0.037, ω_1_ = 1.00, ω_2a_ = 0.037, ω_2b_ = 1.00 Foreground: ω_0_ = 0.037, ω_1_ = 1.00, ω_2a_ = 1.00,ω_2b_ = 1.00	Test 1	-13496.27	0.019	10G, 13G, 62A, 66L, 95V, 188D, 270V, 349E, 352R, 375E, 534V, 578L, 591V
Model A (null hypothesis) for Old World fruit bats	59	*P*_0_ = 0.862, *P*_1_ = 0.090, *P*_2a_ = 0.044,*P*_2b_ = 0.005 Background: ω_0_ = 0.037, ω_1_ = 1.00, ω_2a_ = 0.037, ω_2b_ = 1.00 Foreground: ω_0_ = 0.037, ω_1_ = 1.00, ω_2a_ = 1.00,ω_2b_ = 1.00	Test 2	-13496.27	1	Not allowed
Model A (alternative hypothesis) for New World fruit bats	60	*P*_0_ = 0.837, *P*_1_ = 0.087, *P*_2a_ = 0.068,*P*_2b_ = 0.007 Background: ω_0_ = 0.038, ω_1_ = 1.000, ω_2a_ = 0.038, ω_2b_ = 1.00 Foreground: ω_0_ = 0.038, ω_1_ = 1.00, ω_2a_ = 1.00,ω_2b_ = 1.00	Test 1	-13498.58	0.191	Not allowed
Model A (null hypothesis) for New World fruit bats	59	*P*_0_ = 0.837, *P*_1_ = 0.087, *P*_2a_ = 0.068,*P*_2b_ = 0.007 Background: ω_0_ = 0.038, ω_1_ = 1.00, ω_2a_ = 0.038, ω_2b_ = 1.00 Foreground: ω_0_ = 0.038, ω_1_ = 1.00, ω_2a_ = 1.00,ω_2b_ = 1.00	Test 2	-13498.58	1	Not allowed

^a^np, number of parameters.

^b^LRT, likelihood ratio test.

^c^location of sites with elevated omega values detected by branch-site model A test refer to *Homo sapiens* sequence.

The sites with BEB posterior probabilities > 0.95 were highlight by underline.

**Fig 2:**
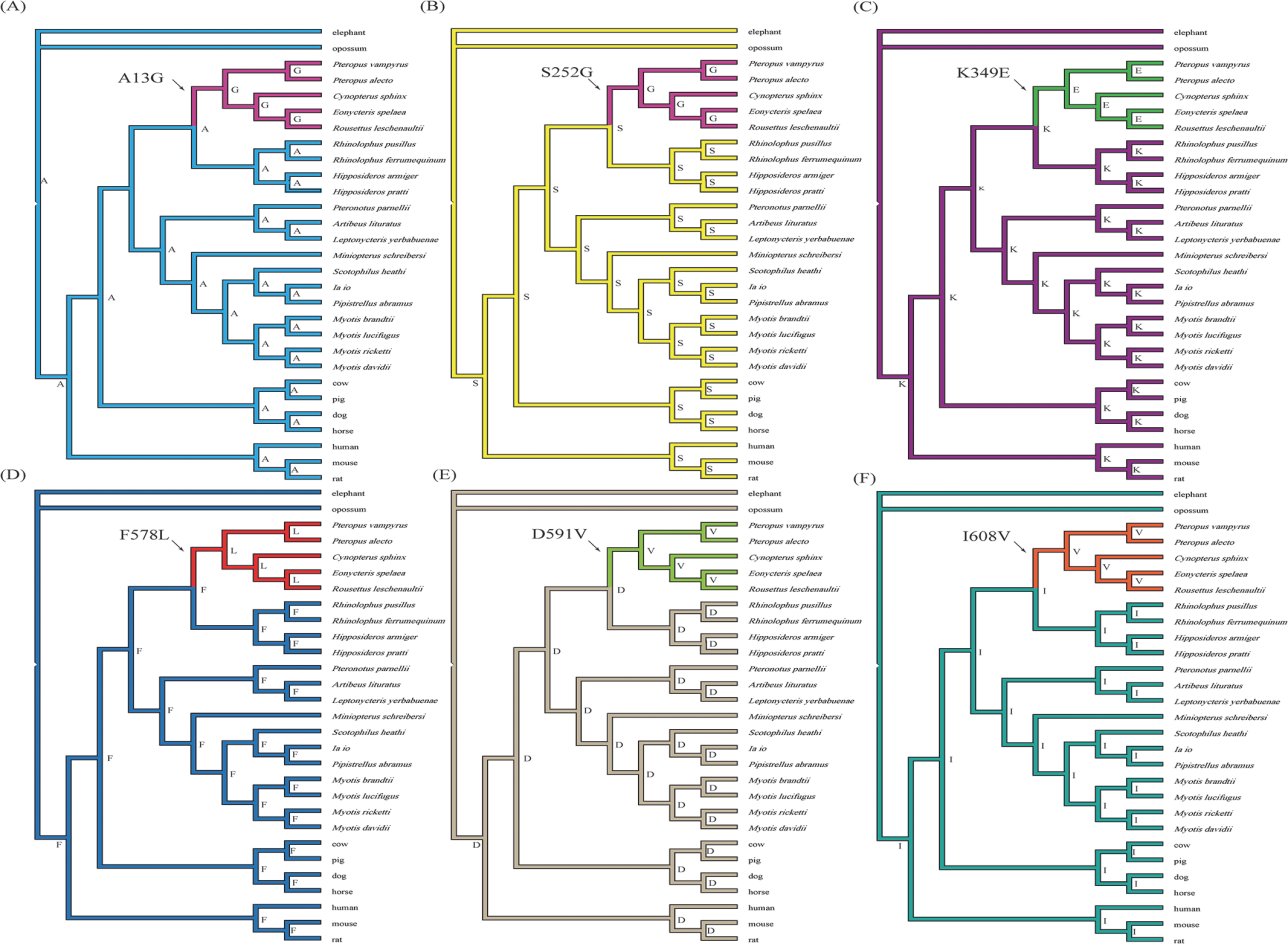
The species tree of 29 mammals with Old World fruit bat specific amino acid replacements identified by ancestral sequence reconstruction using the maximum parsimony method. (A) A13G, (B) S252G, (C) K349E, (D) F578L, (E) D591V and (F) I608V. Branch lengths are not drawn to scale.

**Fig 3:**
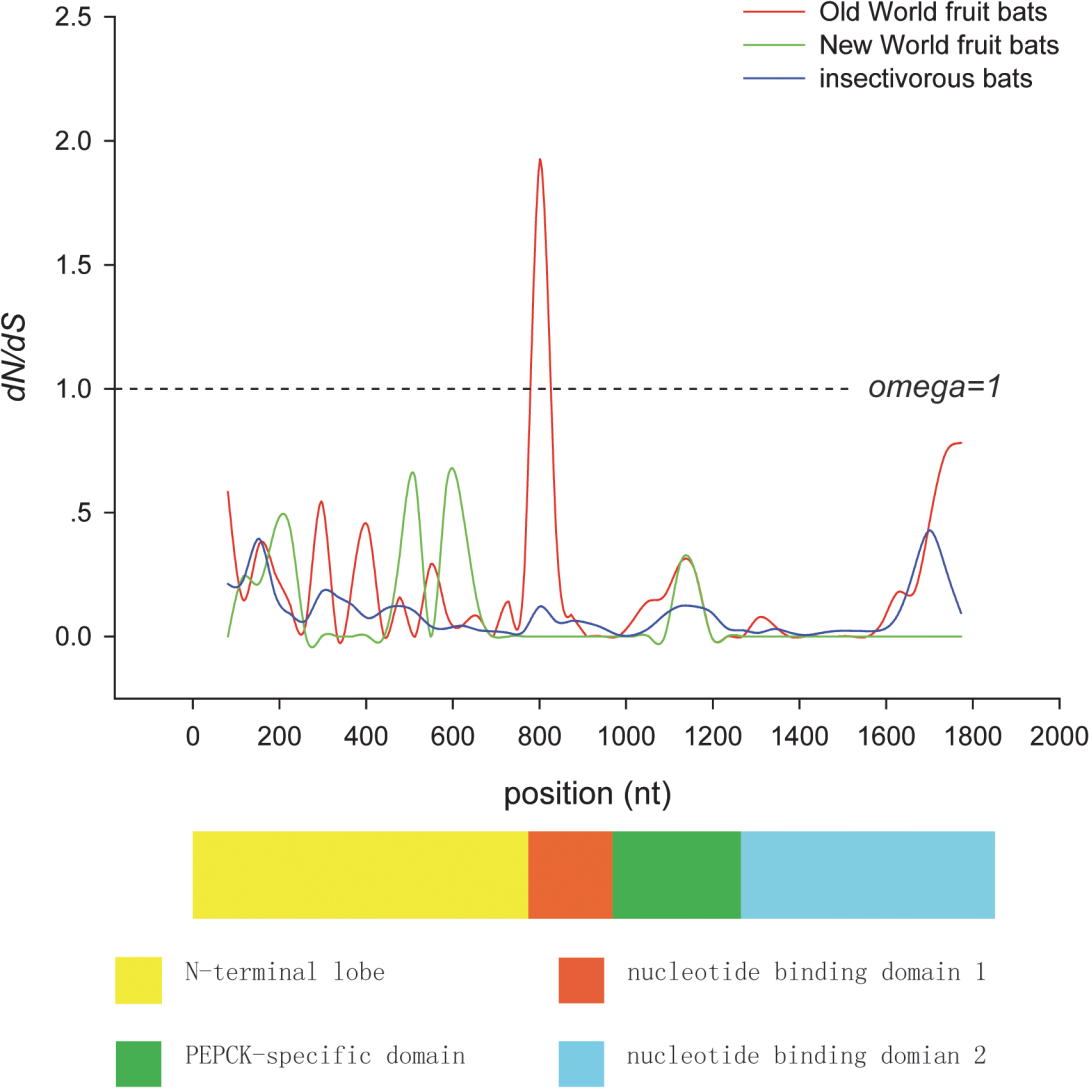
Sliding window analysis of the variation in omega values along *Pck1* genes in OWFBs, NWFBs, and insectivorous bats. Window size and the step size were set to 90bp and 36bp, respectively. Schematic of the PEPCK1 protein domain structure is shown beneath the plot.

To identify amino acid changes on particular branches, we use the software Mesquite 2.74 [48] to reconstruct the ancestral amino acid sequences. These reconstructions showed that there were six specific amino acid replacements (A13G, S252G, K349E, F578L, D591V, and I608V) on the ancestral branch of OWFBs (Fig. 2), and four (A13G, K349E, F578L, and D591V) of them were detected by branch-site model A as having elevated omega values (Table 3). To further explore the selection pressure changes among *Pck1* codons, we calculated the values of ω among OWFBs, NWFBs, and insectivorous bats using SWAAP 1.0.2. The results of this analysis showed that the estimated ω values for OWFBs are much larger than those of insectivorous bats, with a maximal ω value of 1.9 observed on the nucleotide binding domain 1 (Fig. 3). The ω values of the NWFBs were also larger than those of the insect-eating bats, but not as large as those of the OWFBs.

**Fig 4:**
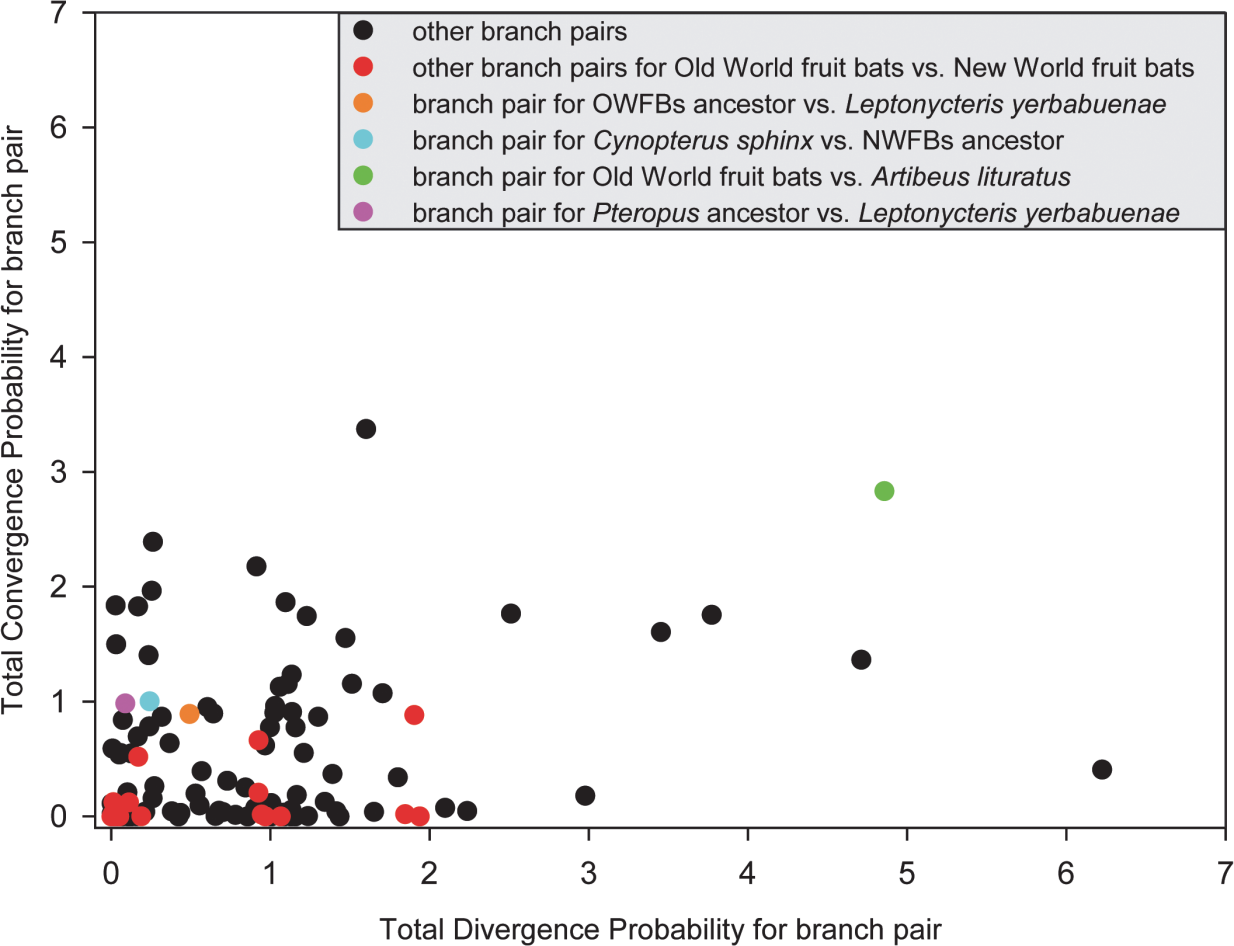
Plot of the total posterior probabilities of divergence versus convergence for all pairs of branches in the tree. Pairwise comparison for the ancestral branch of Old World fruit bats versus *Artibeus lituratus*, the ancestral branch of NWFBs versus *Cynopterus sphinx*, ancestral branch of *Pteropus* versus *Leptonycteris yerbabuenae*, and the ancestral branch of OWFBs versus *Leptonycteris yerbabuenae* are highlighted by green, blue, purple and orange, respectively.

To examine parallel and/or convergent amino acid substitutions between the OWFB and NWFB ancestral branches, we reconstructed ancestral sequences using PAML [43], with the amino acid replacements on the major branches of the fruit-eating bats inferred. Five parallel amino acid substitutions were shared between OWFBs and NWFBs (N127S, Q276R, R503H, I558V, and Q593R). The total posterior probabilities of divergence versus convergence for focus branch-pairs were shown in Fig. 4. We used two methods to test the probability of acquiring the observed parallel substitutions in the frugivorous branches by chance. Both methods suggest that the probabilities of the substitutions Q276R and I558V, shared between the ancestral branches of OWFBs and *A*. *lituratus*, the Q593R substitution, shared between the ancestral branch of NWFBs and *C*. *sphinx*, and the R503H substitution, shared between the ancestral branch of the genera *Pteropus* and *L*. *yerbabuenae*, all deviated significantly from the random expectation (Table 4 and Table 5). The N127S substitution, shared between the ancestral branches of OWFBs and *L*. *yerbabuenae*, however, was not significant (*P* > 0.05), suggesting that this amino acid substitution likely occurred in parallel by chance (Table 4 and Table 5).

**Table 4:** Total probabilities of convergence for parallel amino acid substitutions in the *Pck1* gene for key pairs of branches of frugivorous bats.

Branch Pair^a^	Total Convergence Probability	Parallel Substitutions with BPP Values^b^	***P***-value^c^
1 vs. 5	2.834	Q276R (0.873), I558V (0.996)	0.007
2 vs. 4	1.003	Q593R (0.977)	0.025
3 vs. 6	0.983	R503H (0.983)	0.033
1 vs. 6	0.892	N127S (0.887)	0.229

^a^See Fig. 1(B) for branch labels.

^b^BPP, Bayesian posterior probability.

^c^Significance levels of the branch-wise convergences are comparisons of the observed probabilities against the null distribution based on 1000 simulated data sets.

**Table 5:** Statistical tests for parallel evolution between Old World fruit bats and New World fruit bats.

Branch pair^a^	Parallel substitution	Observed number	Expected number	***P***-value^b^
1 vs. 5	Q276R, I558V	2	0.0307	<0.01
2 vs. 4	Q593R	1	0.0075	0.030
3 vs. 6	R503H	1	0.0096	0.038
1 vs. 6	N127S	1	0.0257	0.103

^a^Selected branches are labeled with Arabic numerals in Fig. 1(B)

^b^The p value of each test was multiplied by four (branch pairs) to correct for multiple testing.

## Discussion

In this study, we examined the molecular evolution of the *Pck1* gene, which plays an important role in the maintenance of glucose homeostasis during fasting in frugivirous bats, which eat carbohydrate-rich diets. As fasting is a regular part of the life cycle of fruit eating bats, they require an enhanced ability to resist hypoglycemia [24,25]. By comparing *Pck1* sequences among frugivorous and insectivorous bats, and other mammals, we provide the first data on the molecular evolution of this gene in frugivorous bats. We found that the omega value (d_N_/d_S_) on the ancestral branch of OWFBs is higher than for the remaining branches of the species tree, and that the rate of nonsynonymous substitutions in the *Pck1* gene of OWFBs is significantly higher than that of insectivorous bats. Although there was a change in the selection pressure acting on the *Pck1* gene on the ancestral branch of NWFBs, the omega value is still quite low indicating that *Pck1* is still under purifying selection.

Although the results of test1 of the branch-site model A test revealed 13 amino acid replacement with elevated omega value, we are unable to rule out the possibility that these were due to a relaxation of the selection acting on the *pck1* gene, as the result of the explicit test of positive selection (test2) was negative. Test1, by itself, is unable to distinguish between relaxation of constraints and positive selection [46]. However, the likelihood that *Pck1* experienced relaxed selection in Old World fruit bats is probably low as PEPCK1 has a very important function across mammals [56]. Even between bacteria and mammals, a number of residues at the active site are conserved and the reaction mechanism for this enzyme in both bacteria and mammals is thought to be same [57]. From our sequenced *Pck1* genes we found that the crucial residues in the active site loops, including the R-loop, P-loop and Ω-loop, are completely conserved. These results indicated that PEPCK1 retains an important role in glucose homeostasis in frugivore bats [58]. Previous studies have shown that knockout of *Pck1* in mice leads to hypoglycemia and death [14], supporting the necessity of PEPCK for life in mammals, and making it unlikely that this gene in OWFBs underwent relaxed evolution. All these results indicate that *Pck1* has experienced strong purifying selection not only in fruit bats, but also in all other mammalian species examined. Although the results are congruent with the fact that the PEPCK1protein is very critical for hepatic glycometabolism, there are two possible explanations that may explain the failure to detect positive selection. First, OWFBs diverged from the other Yinpterochiroptera lineages nearly 60 million years ago [40], and previous studies have shown that signals of positive selection can be swamped by a history of purifying selection [46]. Second, the low level of sequence divergence among the sequences limits the power of statistical methods to detect signals of positive selection [59–61]. In addition, we only had two NWFB species, which further limits our ability to detect positive selection in this group.

**Fig 5:**
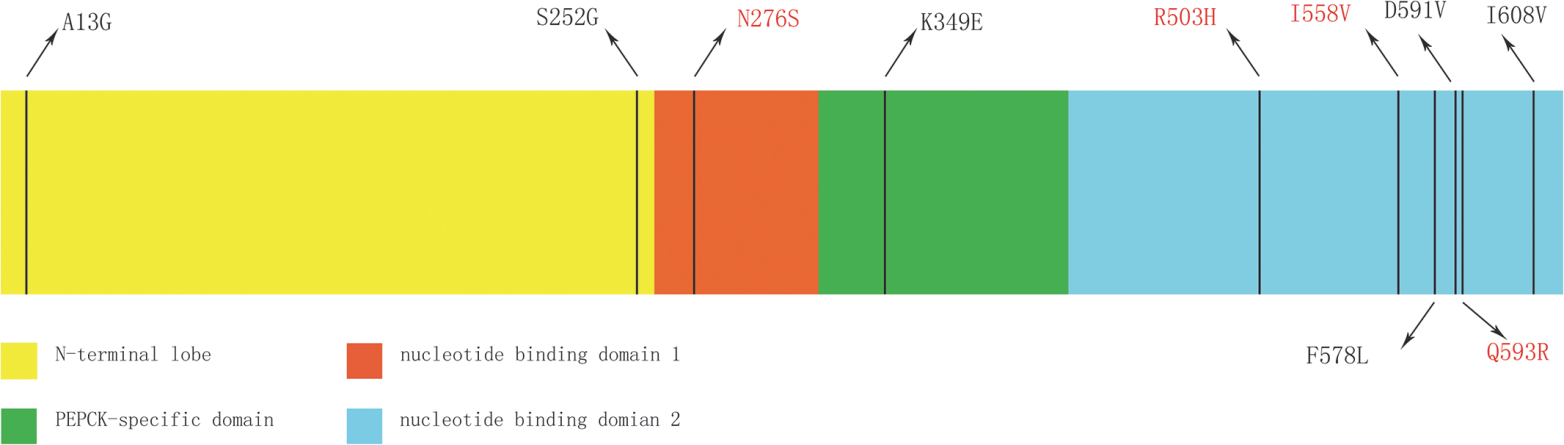
Distribution of the six OWFBs-specific amino acid substitutions and four parallel amino acid substitutions occurring between OWFBs and NWFBs in the secondary structure of PEPCK1 protein. OWFB substitutions are labeled in black, while parallel substitutions are labeled in red within a model of the domain structure of PEPCK1.

Results from the ancestral reconstruction of the amino acid sequences using the maximum parsimony method with Mesquite 2.74 [48] identified six specific amino acid replacement that occur on the OWFBs ancestral lineage. A change from the basic amino acid lysine to the acidic amino acid glutamic acid (K349E) is located in the PEPCK-specific active site domain (residues 326–425) [54]. The A13G and S252G substitutions occur in the N-terminal domain (residues 1–259) associated with degradation and acetylation of PEPCK1 [62]. F578L, D591V and I608V substitutions lie near the nucleotide-binding domain 2 (residues 426–622) (Fig. 5) responsible for GTP binding, which is necessary for enzymatic activity [55]. Sliding window analysis also found a significantly elevated omega value for the sequences encoding the nucleotide-binding domain 1 (residues 260–325). The elevated omega value is likely due to the I270V substitution, which was detected by the branch-site model A. Replacement of I270V is a OWFBs-specific change among species of the order Chiroptera, and is only shared with the pig (data not shown).

In contrast to OWFBs, we did not find any specific amino acid replacements in the NWFBs. It is interesting to note that other recent molecular evolution studies focusing on diet-related genes in bats also found similar patterns [63–65]. For example, GLUT4 which play an important role in glucose homeostasis had two specific amino acid substitutions only in the OWFBs but not in NWFBs [64]. This evolutionary discrepancy in patterns of sequence evolution between OWFBs and NWFBs for the *Pck1* gene may have two explanations. First, OWFBs evolved their carbohydrate-rich diet earlier than the NWFBs (28 mya and 20 mya, respectively) [40], thus have had a greater amount of time to accumulated accelerated changes. Second, the specific sequence changes may happen in the regulatory regions in NWFBs, instead of the coding region of these genes, as the promoter can regulate the expression of PEPCK1 [66], change may only occur at the protein abundance and not the protein sequence level.

Although no evidence for positive selection was found in the *Pck1* gene of OWFBs and NWFBs, this does not mean that this gene did not undergo adaptive evolution. Parallel evolution between OWFBs and NWFBs is also a strong indicator of adaptive evolution [53]. Previous molecular evolution studies in bats have also found significant evidence for parallel or convergent evolution with no evidence for positive selection [67,68]. A possible explanation for the parallel evolution at the amino acid level between *Pck1* genes in OWFBs and NWFBs is molecular adaption to the regular fasting by these fruit bats. It is widely accepted that the hepatic gluconeogenesis plays a critical role in the maintenance of glucose homeostasis during fasting [5,8], and it has been shown that hepatic gluconeogenesis is the primary metabolic mechanism for fasting resistance in the Old World Fruit Bats [31]. PEPCK1, the key enzyme of gluconeogenesis, has been shown in a previous study to have a crucial role in fasting in New World fruit bats [32]. Thus, it is reasonable to assume that the parallel evolution of *Pck1* in OWFBs and NWFBs might relate to the evolution of adaptive mechanisms for fasting resistance in fruit bats. Here we found four credible parallel evolution sites (Q276R, R503H, I558V and Q593R) in PEPCK1. All of these sites are located near the nucleotide binding domain, which might influence the binding of GTP to PEPCK1 and thus enzymatic activity [54] (Fig. 5). It is interesting to note that, besides the four convergently evolving sites, the Old World fruit bats also have six other specific amino acid substitutions, which may reflect the fact that the evolutionary history of OWFBs is longer than that of the NWFBs [40].

In conclusion, our results show that the *Pck1* gene, which plays an important role in the maintenance of glucose homeostasis during fasting, has undergone parallel evolution between the OWFBs and NWFBs, which provides evidence for adaptive evolution in both lineages, although our evidence for NWFBs is limited to only two species of Phyllostomid bats. Greater sampling of NWFBs is needed to confirm this finding. Apart from gluconeogenesis, PEPCK1 is also the rate-limiting enzyme in glyceroneogenesis, which is responsible for recycling free fatty acid back to triglyceride [69]. Chronic release of free fatty acid by adipose tissue is a critical factor in the development of Type 2 diabetes [70], therefore, PEPCK1 could prevent diabetes by controlling the rate of glyceroneogenesis [70]. Here we found evidence for parallel evolution of the *Pck1* gene between OWFBs and NWFBs, which could possibly be explained by its function in glyceroneogenesis. However, additional in-depth studies are needed to address this issue. We speculate that additional deeper studies will reveal that numerous other genes related to carbohydrate metabolism will be found to have experienced adaptive evolution in OWFBs and NWFBs.

## Supporting Information

S1 Fig Enzymatic pathway for gluconeogenesis. Four unique enzymes required in gluconeogenesis are shown in the rectangles. All of the remaining steps are catalyzed by glycolytic enzymes.
(TIF)

S1 Table Information on species examined in the study. (DOC)

S1 Text Details on some methods used in this study. (DOC)
