# Supplementary figures and images for: Phosphoenolpyruvate Carboxykinase 1 Gene (Pck1) Displays Parallel Evolution between Old World and New World Fruit Bats
Source: PLoS One. 2015 Mar 25;10(3):e0118666. doi: 10.1371/journal.pone.0118666 (PMC4373879; doi:10.1371/journal.pone.0118666)

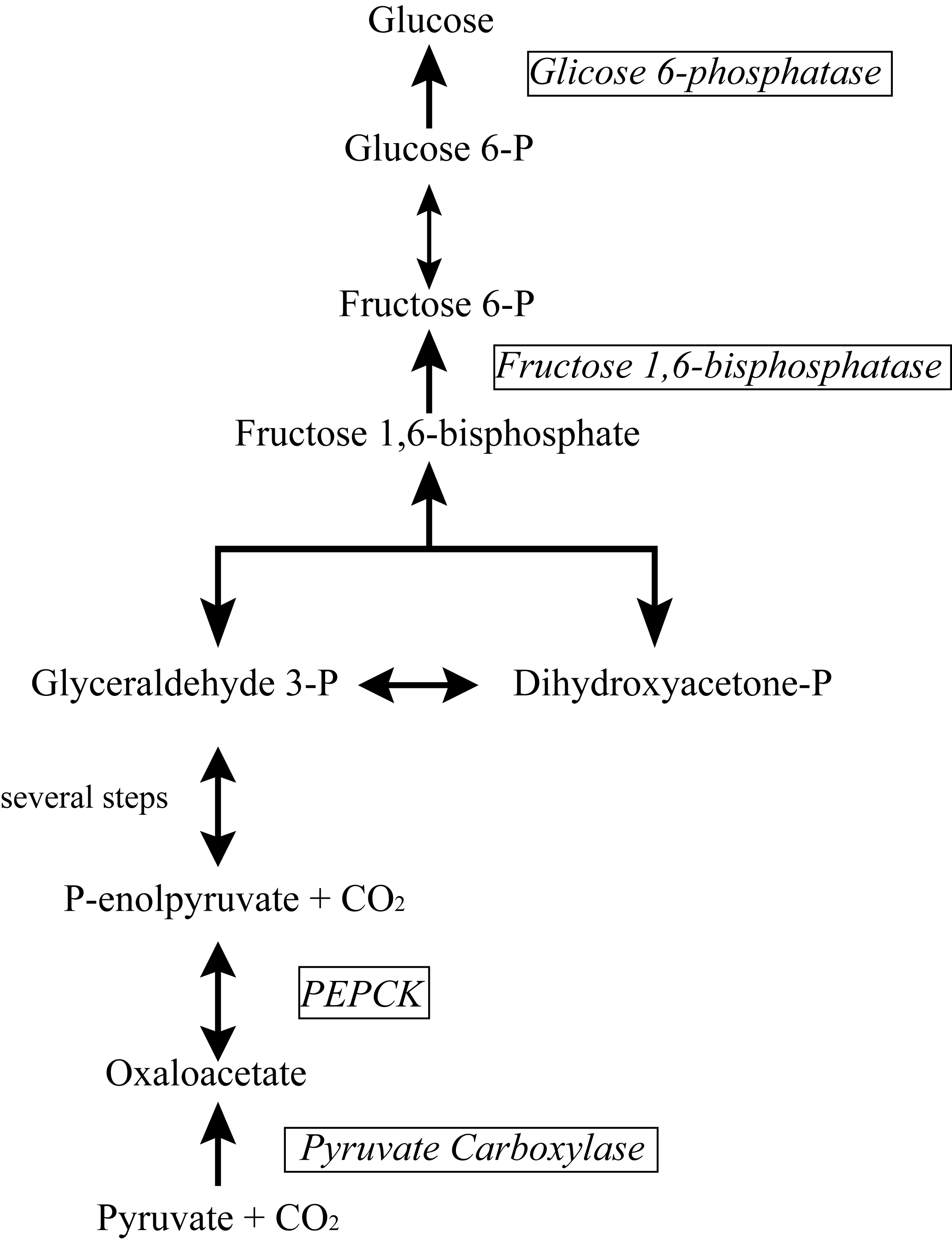

Supplement: S1 Fig — Four unique enzymes required in gluconeogenesis are shown in the rectangles. All of the remaining steps are catalyzed by glycolytic enzymes. (TIF) [file pone.0118666.s001.tif]
