# Supplementary material for: Phosphoenolpyruvate Carboxykinase 1 Gene (Pck1) Displays Parallel Evolution between Old World and New World Fruit Bats
Source: PLoS One. 2015 Mar 25;10(3):e0118666. doi: 10.1371/journal.pone.0118666 (PMC4373879; doi:10.1371/journal.pone.0118666)
Supplement: S1 Table — (DOC) [file pone.0118666.s002.doc]

**Table S1. Information on species examined in the study.**

| Species Name | Order | Accession No. |
| --- | --- | --- |
| *Pteropus vampyrus* | Chiroptera | ENSPVAG00000007382 |
| *Pteropus alecto* |  | XM_006922098 |
| *Cynopterus sphinx* |  | KJ957749 |
| *Eonycteris spelaea* |  | KJ957750 |
| *Rousettus leschenaultii* |  | KJ957751 |
| *Rhinolophus pusillus* |  | KJ957752 |
| *Rhinolophus ferrumequinum* |  | KJ957753 |
| *Hipposideros armiger* |  | KJ957754 |
| *Hipposideros pratti* |  | KJ957755 |
| *Pteronotus parnellii* |  | KJ957756 |
| *Artibeus lituratus* |  | KJ957757 |
| *Leptonycteris yerbabuenae* |  | KJ957758 |
| *Miniopterus schreibersi* |  | KJ957759 |
| *Scotophilus heathi* |  | KJ957760 |
| *Ia io* |  | KJ957761 |
| *Pipistrellus abramus* |  | KJ957762 |
| *Myotis ricketti* |  | KJ957763 |
| *Myotis davidii* |  | XM_006758839 |
| *Myotis brandtii* |  | XM_005880675 |
| *Myotis lucifugus* |  | XM_006095420 |
| *Homo sapiens* | Primates | NM_002591 |
| *Mus musculus* | Rodentia | NM_011044 |
| *Rattus norvegicus* |  | NM_198780 |
| *Equus caballus* | Perissodactyla | XM_001489771 |
| *Bos taurus* | Cetartiodactyla | NM_174737 |
| *Sus scrofa* |  | NM_001123158 |
| *Canis familiaris* | Carnivora | NM_001197143 |
| *Loxodonta Africana* | Proboscidea | XM_003419943 |
| *Monodelphis domestica* | Didelphimorphia | XM_001377770 |
