# Supplementary material for: Phosphoenolpyruvate Carboxykinase 1 Gene (Pck1) Displays Parallel Evolution between Old World and New World Fruit Bats
Source: PLoS One. 2015 Mar 25;10(3):e0118666. doi: 10.1371/journal.pone.0118666 (PMC4373879; doi:10.1371/journal.pone.0118666)
Supplement: S1 Text — (DOC) [file pone.0118666.s003.doc]

**Supporting Information Text**

**Details on some methods used in this study**

Differences in rates of synonymous and nonsynonymous substitutions

“PairwiseRelativeRate.bf” from the HyPhy package [1] was used to detect significant differences in the rates of synonymous and nonsynonymous substitutions between the frugivorous and insectivorous bats. For this analysis, the opossum served as the outgroup, based on the accepted species tree, and differences were determined for all pairs of species between the frugivorous and insectivorous bats using the Muse-Gaut 94 codon model [2].

Detection of parallel and convergent substitutions

To detect evidence of parallel or convergent amino acid substitutions between OWFBs and NWFBs, we used a method described by Castoe et al. implemented in the codeMLancestral package [3]. In this analysis, site-wise convergent and divergent posterior probabilities for all possible amino acid substitutions were estimated along each branch of the species tree under the JTT amino acid substitutions model. Branch lengths for the species tree were estimated using MrBayes 3.1.2 [4]. To access the probabilities of convergence and divergence for all pairwise branch comparisons, the probabilities of all pairs of convergent substitutions and divergent substitutions for each branch were summed. We compared the branch pairs of the OWFBs versus the NWFBs with the other branch pairs.

Testing convergence between sequences

To test whether the convergence detected between pairs of focal branches was significant, we used a method described by Rossiter et al [5]. In this analysis, we compared the observed probabilities against a null distribution based on simulations. First, we generated 1000 simulated *Pck1* gene sequences using EVOLVER [6] with the JTT amino acid substitution model and the accepted species tree topology. The branch-wise convergence probabilities of the 1000 replicates were then calculated using codeMLancestral package [3]. The convergence probabilities from the observed data were then compared to the distribution from the 1000 simulated sequences.

1. Pond SLK, Muse SV (2005) HyPhy: hypothesis testing using phylogenies. Statistical methods in molecular evolution: Springer. pp. 125-181.

2. Muse SV, Gaut BS (1994) A likelihood approach for comparing synonymous and nonsynonymous nucleotide substitution rates, with application to the chloroplast genome. Molecular Biology and Evolution 11: 715-724.

3. Castoe TA, de Koning AJ, Kim H-M, Gu W, Noonan BP, et al. (2009) Evidence for an ancient adaptive episode of convergent molecular evolution. Proceedings of the National Academy of Sciences 106: 8986-8991.

4. Ronquist F, Huelsenbeck JP (2003) MrBayes 3: Bayesian phylogenetic inference under mixed models. Bioinformatics 19: 1572-1574.

5. Davies K, Cotton J, Kirwan J, Teeling E, Rossiter S (2012) Parallel signatures of sequence evolution among hearing genes in echolocating mammals: an emerging model of genetic convergence. Heredity 108: 480-489.

6. Yang Z (2007) PAML 4: phylogenetic analysis by maximum likelihood. Molecular Biology and Evolution 24: 1586-1591.
